# Coexistence of mal de Meleda and congenital cataract in a consanguineous Tunisian family: two case reports

**DOI:** 10.1186/1752-1947-4-108

**Published:** 2010-04-20

**Authors:** Mbarka Bchetnia, Ahlem Merdassi, Cherine Charfeddine, Fatma Mgaieth, Selma Kassar, Farah Ouechtati, Ibtissem Chouchene, Hamouda Boussen, Mourad Mokni, Amel Dhahri-Ben Osman, Med Samir Boubaker, Sonia Abdelhak, Leila Elmatri

**Affiliations:** 1 Molecular Investigation of Genetic Orphan Diseases Research Unit, Institut Pasteur de Tunis, Tunis, Tunisia; 2 Hereditary Keratinization Disorders Research Unit, Hôpital de la Rabta de Tunis, Tunis, Tunisia; 3 Oculogenetic Research Unit, Hôpital Hedi Rais Tunis, Tunis, Tunisia; 4 Department of Pathology, Institut Pasteur de Tunis, Tunis, Tunisia; 5 Department of Medical Oncology, Institut Salah Azaiz, Tunis, Tunisia; 6 Department of Dermatology, Hôpital La Rabta, Tunis, Tunisia

## Abstract

**Introduction:**

Mal de Meleda is a rare form of palmoplantar keratoderma, with autosomal recessive transmission. It is characterized by diffuse erythema and hyperkeratosis of the palms and soles. Recently, mutations in the ARS (component B) gene (ARS, MIM: 606119) on chromosome 8q24.3 have been identified in families with this disorder. Congenital cataract is a visual disease that may interfere with sharp imaging of the retina. Mutations in the heat-shock transcription factor 4 gene (HSF4; MIM: 602438) may result in both autosomal dominant and autosomal recessive congenital cataracts.

**Case presentation:**

A Tunisian family with two female siblings aged 45 and 30 years, presented with a clinical association of mal de Meleda and congenital cataract. The two patients exhibited diffuse palmoplantar keratodermas. One of them presented with a total posterior subcapsular cataract and had a best corrected visual acuity at 1/20 in the left eye and with the right eye was only able to count fingers at a distance of one foot. The other woman had a slight posterior subcapsular lenticular opacity and her best corrected visual acuity was 8/10 in the right eye and with her left eye she was only able to count fingers at a distance of one foot. A mutational analysis of their ARS gene revealed the presence of the homozygous missense mutation C99Y and two single nucleotide polymorphisms (-55G>C and -60G>C). The splice mutation (c.1327+4A-G) within intron 12 of the HSF4 gene, which has been previously described in Tunisian families with congenital cataract, was not found in the two probands within this family.

**Conclusion:**

To the best of our knowledge, such original clinical association has not been reported previously. The association of these two autosomal recessive diseases might have occurred in this family due to a high degree of inbreeding. The C99Y mutation may be specific to the Tunisian population as it has been exclusively reported so far in only three Tunisian families with mal de Meleda.

## Introduction

Keratosis palmoplantaris transgrediens of Siemens or mal de Meleda (MdM, MIM: 248300) is a rare autosomal recessive genodermatosis. It has a prevalence of one in 100,000 in the general population [1]. MdM is characterized clinically by erythema and hyperkeratosis of the palms and soles which have a sharp demarcation and that progress with age (known as progrediens) and extend to the dorsal aspects of the hands and feet (known as transgrediens). Its histological features include acanthosis, hyperkeratosis, and foci of parakeratosis [2]. MdM is due to mutations in the ARS (component B) gene encoding the SLURP-1 (secreted Ly-6/uPAR related protein-1) protein. SLURP-1 is a member of the Ly-6/uPAR protein family and homologous to snake venom and frog neurotoxins [3].

Congenital cataract is a major eye abnormality and often leads to blindness in babies [4]. Non-syndromic familial cataracts are usually inherited as a dominant trait, while the autosomal recessive and X-linked forms are less common [5]. More than 20 genes or loci have been identified for autosomal dominant cataracts. However, only three loci, at 3p, 9q13-q22 and 19q13 [6-8], and four genes (CRYAA, LIM2, GCNT2 and HSF4) have been implicated in autosomal recessive cataracts [9-12].

We report a clinical and genetic investigation of two patients from a Tunisian family affected by MdM and congenital cataracts.

## Case presentation

**Figure 1:**
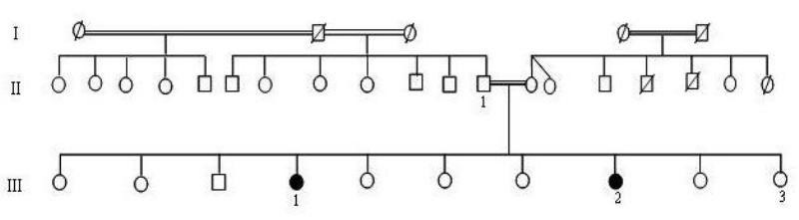
**Pedigree of the family with mal de Meleda and congenital cataract**. Solid symbols represent affected individuals with mal de Meleda and congenital cataract. Open symbols represent unaffected individuals.

**Figure 2:**
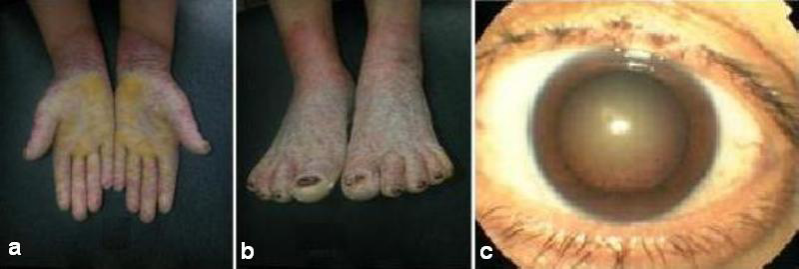
**Clinical manifestations of the Tunisian patients with mal de Meleda**. (A) and (B) Transgressive keratodermas and palmoplantar erythema resulting in the characteristic 'glove and stocking' distribution. (C) Photograph of our patient III-1 with a total posterior subcapsular cataract.

A consanguineous family that originated from Tunisia was investigated for MdM (Figure 1). Four available members identified as II-1, III-1, III-2 and III-3, aged 75, 45, 30 and 26 years, respectively, underwent clinical examination by at least one dermatologist to check for a cutaneous disease and by an ophthalmologist for an ocular disorder. Individuals III-1 and III-2 (our female probands) presented with the clinical association of MdM and/or congenital cataracts. There were no other family members with MdM or congenital cataracts over three generations. Patients III-1 and III-2 presented with diffuse keratodermas and hyperhidrosis involving the palms and soles. The onset of MdM and congenital cataract occurred during the first months of the lives of the affected patients. The mode of transmission of the MdM phenotype was of an autosomal recessive nature. The keratoderma extended in a transgrediens fashion and progressed with age, thus resulting in the characteristic 'glove and stocking' distribution (Figures 2A and 2B). A histological examination of their MdM lesions revealed hypergranulosis, hyperkeratosis, parakeratosis, and moderate acanthosis. Focal spongiosis and mononuclear exocytosis were also noted. Patient III-3 did not show the apparent clinical manifestations of the MdM phenotype.

The father (II-1) of these siblings was affected by multiple extended lesions of his thorax and limbs, with a histological diagnosis of human immunodeficiency virus (HIV)-negative Kaposi's sarcoma confined to his skin since 2004. The disease was clinically severe with bulky and significant lesions covering more than 50% of the surface of the affected areas. Laboratory work-up for extracutaneous (lung, digestive tract and ear-nose-throat) lesions was negative. He was intermittently treated with oral and intravenous etoposide, which resulted in minor clinical responses. He died as a result of disease progression and renal failure in July 2008.

Congenital cataract was transmitted as an autosomal recessive trait in this family. The difference in lens density between the two eyes was documented in both patients III-1 and III-2. Patient III-1 presented with a total posterior subcapsular cataract. Her best corrected visual acuity was 1/20 in the left eye and with the right eye was only able to count fingers at a distance of one foot (Figure 2C). Meanwhile, patient III-2 had a slight posterior subcapsular lenticular opacity. Her best corrected visual acuity was 8/10 in the right eye and with her left eye she was only able to count fingers at a distance of one foot.

After obtaining informed consent, genomic DNA was extracted from peripheral blood leukocytes of our patients using standard procedures. We designed intronic oligonucleotide primers, which flanked the coding exons of their ARS gene according to the public genome sequence (accession number X99977). For the HSF4 gene, we designed primers simultaneously, amplifying exons 12 and 13 in order to test the recurrent splice site mutation (c.1327+4A-G). A polymerase chain reaction (PCR) test was carried out as described by Charfeddine *et al*. [13]. The PCR template was sequenced using an ABI 3130 automated sequencing system (Applied Biosystems, California, USA).

**Figure 3:**
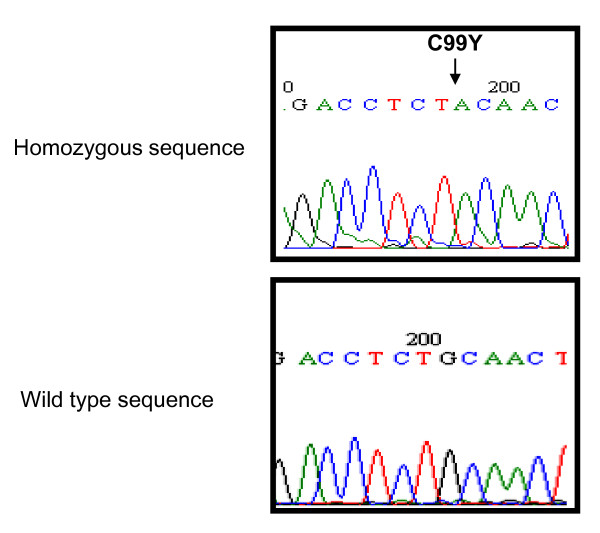
**Genomic DNA sequence of exon 3 of the ARS gene for patient III-1 that shows the G to A homozygous missense substitution at nucleotide 296, which leads to an amino acid change from cysteine to tyrosine at codon 99 (C99Y) (upper panel)**. Wild-type sequence of unrelated control (lower panel).

A mutation screening of the affected family members (III-1 and III-2) revealed the presence of a homozygous G-A transition at nucleotide 296 in exon 3, thus predicting a conversion of cysteine to tyrosine at amino acid 99 (C99Y) (Figure 3). We also found two additional sequence variations both corresponding to a transversion from C to G at a homozygous state, respectively at positions 55 and 60 nucleotides upstream of the ATG start codon in exon 1. These neutral polymorphisms were not disease-associated, as they were present in both healthy and affected individuals. The father and the non-affected daughter (III-3) were heterozygous for the C99Y missense mutation.

The molecular investigation of the congenital cataract in the affected family members by screening the splice variation (c.1327+4A-G) in the HSF4 gene showed the absence of this mutation.

## Discussion

The association of ocular manifestations with MdM has already been described by Durmus *et al*. in a case presenting with bilateral macular deposits and the MdM phenotype [14]. To the best of our knowledge, the coexistence of both MdM and congenital cataract in the same patient had not been reported previously. This clinical association is likely to be accidental as probands were born to a consanguineous mating, and the two diseases are transmitted as an autosomal recessive trait.

Patient III-3 was heterozygous for the C99Y mutation. We previously reported that female heterozygous carriers of several ARS gene mutations express attenuated signs of skin disease [15]. We did not, however, observe such skin changes in our heterozygous patient III-3. It is noteworthy that her father suffered from cutaneous cancer. Taking into account the rarity of the studied phenotype, it is not possible to hypothesize on the influence of his status as a heterozygous carrier of an MdM mutation, as well as on the severity of the disease.

Although the C99Y mutation is at the N terminus of the protein, it affects cysteine residues implicated in one of the five highly conserved disulfide bridges of the SLURP-1 protein. It alters a cysteine contained in a consensus carboxy-terminal sequence shared by all family members with the Ly-6/uPAR gene, such as in snake and frog cytotoxins. To date, this variation seems to be specific to Tunisian families with MdM as it has not been reported in other populations and may implicate a common ancestral allele.

Taking into account previous studies showing genetic and mutation homogeneity of genetic diseases in Tunisia [16,17], the splice variation (c.1327+4A-G) in the HSF4 gene previously identified in one Tunisian family with an autosomal recessive cataract was screened in individuals with MdM and cataract. We have not found this mutation in the family described in this case report. Further genetic analyses are needed to confirm or exclude the involvement of the HSF4 gene or one of the loci or genes reported for autosomal recessive cataracts.

## Conclusion

To the best of our knowledge, the coexistence of both MdM and congenital cataract in the same patient has not been reported previously. The two phenotypes might segregate separately, although their co-occurrence could be more than a coincidental finding as the lens and the skin have the same embryological origins.

## Abbreviations

MdM: mal de Meleda; PCR: polymerase chain reaction; SLURP-1: secreted Ly-6/uPAR related protein-1.

## Consent

Written informed consent was obtained from the patients for publication of this case report and any accompanying images. A copy of the written consent is available for review by the Editor-in-Chief of this journal.

## Competing interests

The authors declare that they have no competing interests.

## Authors' contributions

MB, CC and SK participated in the analysis and interpretation of data. MB wrote the manuscript. AM, FM, FO, IC and HB collected the clinical data. MM, ADO, MSB, SA and LE contributed to the critical revision of the manuscript for significant intellectual content, study concept and design. All authors read and approved the final manuscript.
